# Association between distress and displacement settings: a cross-sectional survey among displaced Yazidis in northern Iraq

**DOI:** 10.1186/s12889-021-10734-8

**Published:** 2021-04-08

**Authors:** Phuong N. Pham, Laila Fozouni, Abdulrazzaq al-Saiedi, Kevin Coughlin, Patrick Vinck

**Affiliations:** 1 Harvard Humanitarian Initiative, Harvard University, 14 Story Street, Cambridge, MA 02138 USA; 2 Harvard T.H. Chan School of Public Health, 677 Huntington Ave, Boston, MA 02115 USA; 3 Brigham and Women’s Hospital, 75 Francis St, Boston, MA 02115 USA; 4 School of Medicine, University of California, San Francisco, 505 Parnassus Ave, San Francisco, CA 94143 USA

**Keywords:** Refugees, Built environment, Housing, Mental health, Trauma

## Abstract

**Background:**

Globally 70.8 million people have been forcibly displaced from their homes and are at disproportionally high risk for trauma. At the time of this study, there was an estimated 1.6 million internally displaced persons (IDP) in Iraq, more than two-thirds of whom reside in private, urban settings. This study aims to understand the impact of post-displacement accommodation on mental well-being of the Yazidi minority group displaced in Iraq.

**Methods:**

Multi-stage stratified sampling was used to randomly select IDPs in camp and out of camp settlements in northern Iraq. Standardized questionnaires evaluated factors including exposure to violence and self-reported distress symptoms (measured by Impact of Event Scale-Revised). A multi-variate linear model assessed the relationship between settlement setting and distress symptoms.

**Results:**

One thousand two hundred fifty-six displaced Yazidi participants were included in the study: 63% in camps and 37% out of camps. After controlling for exposure to violence, social cohesion, unemployment, and access to basic services, IDPs in camps were predicted to have a 19% higher mean distress symptom score compared to those out of camps.

**Conclusions:**

This study provides a framework to investigate post-displacement accommodation as a potential intervention to improve well-being for displaced populations. With a shift towards new models of emergency and long-term housing, it is important to understand the potential and limitations of more decentralized models, and identify effective methods to maintain access to basic services while improving living conditions for both displaced populations and their host communities.

## Background

Worldwide, displaced populations are at a disproportionally high risk for trauma and mental illness such as post-traumatic stress disorder (PTSD), depression, and somatization before, during and after displacement [1–6]. The built environment (i.e. man-made structures in which people live and work) plays an important role in displaced populations’ well-being [7–12]. Research has identified camp conditions and post-displacement accommodations as important predictors of mental health [11, 13]. Exposure to violence and daily stressors, including those associated with the built environment and socioeconomic hardship, are associated with PTSD and poor mental health among these populations, suggesting that discontinuation of these stressors can be an important intervention to mitigate the consequences of trauma [4, 14, 15]. Separately, the relationship between cities, urban living and mental health has been well documented for the general population, but not for displaced populations [16–18]. As displaced populations increasingly seek shelter in urban areas and the international community shifts away from establishing traditional camps for displaced populations, there is a need to better understand the implication of temporary settlement choices on mental health and explore how the built environment specifically can be conceptualized as an intervention to improve mental health and long-term outcomes [11].

The purpose of this study is to compare mental health outcomes and distress symptoms among internally displaced persons (IDPs) living in camps versus out of formal camp settlements in northern Iraq. Understanding and addressing mental health issues among this group is important; poor mental health can cause a myriad of downstream effects, including negatively impacting views on reconciliation and non-violence as a means to end conflict [19, 20].

This study focused specifically on Yazidis, an ethno-religious minority group from northwestern Iraq, which was victim to mass atrocities committed by the Islamic State (IS) [21]. On August 2014, IS attacked the city of Sinjar and its surrounding areas in Nineveh Governate where the Yazidi communities were primarily based. Although IS no longer occupies the Yazidi areas in the Nineveh Governorate (including Sinjar), about 230,000 Yazidis remain displaced in the Kurdistan region [22]. A study of Yazidi women and children, 16% of whom had survived enslavement, found that over 80% met clinical criteria for DSM-5 PTSD diagnosis [23].

At the time of this study, there were an estimated 1.6 million IDPs in Iraq, many of whom were targeted by ISIL in 2013 for their identity, including Sunni and Shi’a Muslims, Shabak, Turkmen, and Yazidis [24, 25]. More than two-thirds of displaced people in Iraq reside out of camps; the majority of IDPs residing out of camps live in rented accommodations, and a smaller percentage live with host families or in informal settlements (e.g. unoccupied buildings) [26–28]. We hypothesized that while IDPs outside of formal camp settlements face several adverse factors [29], they also retain a higher level of control and quality of life, including social interactions, compared to camp-based IDPs, contributing to improved mental health.

## Methods

### Survey sites and sample selection

Participants for this study were selected among IDPs in out of camp (urban) and camp-based settlements in the Niveneh governorate and in the Erbil and Duhok governorates in the Kurdistan Region of Iraq (KRI). Multi-stage stratified sampling strategies were used for both population groups.. Sample size for the two population groups was determined using the sample size formulation for difference in proportions. We assumed a 90% confidence with a Z distribution, 80% power, and a difference of 20% and a design effect of 2, for a total target of 150 interviews per group. This target was adjusted by 20% for anticipated non-response and rounded for logistical purposes and equal assignment to interviewers, for a target of 200 interviews. We anticipated multiple comparisons within each population groups based on gender and known ethnic diversity. The target sample size for IDP in camps was 1800 interviews. The target sample size for IDPs out of camp was 1200.

For IDPs in camps, 15 out of 31 camps for IDPs were randomly selected proportionate to population size using the latest data from the United Nations High Commissioner for Refugees (UNHCR). Within camps, interviewers were randomly assigned to blocks of similar sizes where they used a geographic sampling method to randomly select participants. Interviews could not be completed in two of the camps because the presence of IS family members posed a security risk for the interviewers. As a result, 1575 interviews were ultimately conducted with IDPs in camps (87.5% of targeted 1800 interviews, within the 20% margin).

A similar multi-stage stratified sampling strategy was used to randomly select IDPs out of camps. Five out of 12 subdistricts were randomly selected proportionately to the out of camp population size based on the latest data from the International Organization for Migration (IOM) (mid-2018) available at the time of the survey. Within the subdistricts, 3 sites (neighborhoods) identified by IOM as hosting IDPs were randomly selected, proportionate to the IDP population size. In one subdistrict, four sites were selected due to the small population at each site. Once at the site, interviewers randomly selected a direction and approached every fifth household. If the household did not host IDPs, they were skipped. Because of the small number of IDPs present at each site, an additional 9 sites (neighborhoods) were randomly sampled, resulting in a final sample size of 1406 IDPs not living in camps (117.2% of the target).

From the two sample of IDPs (in and out of camps), only those that indicated being part of the Yazidi ethnic religious group were included in this study. The rationale for the sub-sample was to increase homogeneity in the socio-cultural background and characteristics that have been shown to be associated with mental health. Among 1575 IDPs residing in camp, 794 were Yazidis (50.4%), and 462 Yazidis were among the sampled 1406 IDPs living out of camps (32.9%).

The study protocol was reviewed and approved by Partners Human Research Committee (PHRC, Protocol #2018P001748)) and a local ad-hoc committee and local authorities in northern Iraq where the data were collected.

### Data collection instruments and scale

Respondents were asked questions regarding exposures to violence, social cohesion, access to services, and household assets. Household assets were categorized as medium assets (bed, fridge, and washing machine) and extended assets (mobile phone, internet access, radio, computer, microwave, heater, car, and television) per the UNHCR Vulnerability Assessment [30].

The 22-item Impact of Event Scale-Revised (IES-R) based on a Likert scale of 0 (not at all) to 4 (in full) was used as a validated instrument to estimate subjective distress and calculate a distress score. Possible scores ranged from 0 to 88, and a score of greater than 33 has been established to be have high diagnostic accuracy for PTSD [31, 32].

### Statistical analysis

Statistical analyses were performed using Stata (v15, SE). Baseline demographics were presented as medians [interquartile ranges (IQR)] for continuous variables or percentages for categorical variables and compared by post-migration settlement location using Wilcoxon rank sum or chi-square test. Given low numbers of non-response, non-response answers were treated as missing values.

Linear regression was used to assess associations between distress scores and settlement location. Exposure to violence variables were converted into binary variables in regression to reflect *any* history of past exposure. Social cohesion variables were aggregated into a score (ranging from 1 to 29), with 1 indicating the least amount and 29 the highest amount of social cohesion. All variables associated with the outcome of interest with a *p*-value of < 0.2 in univariable analysis or deemed relevant to post-displacement accommodation were evaluated for inclusion in the final model. Backwards stepwise regression was then performed to derive the final multivariable model which included variables associated with a *p*-value of < 0.05 as well as variables forced in for known association with distress score.

## Results

### General characteristics of respondents

**Fig. 1:**
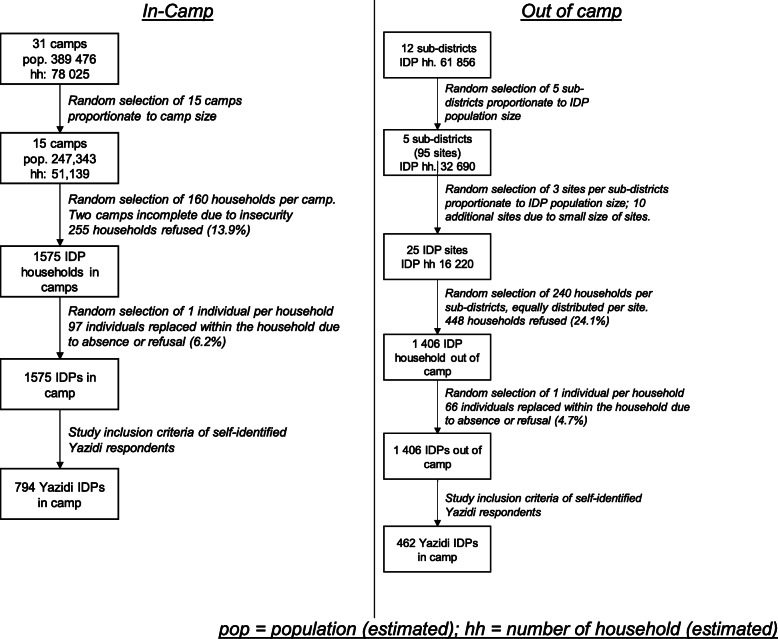
Sampling and stratification strategies. pop = population (estimated); hh = number of household (estimated)

A total of 1256 participants were included in the study; 63% of sampled respondents were living in camps and 37% were living out of camps, all in urban settings, at the time of the survey (Fig. 1).

As shown in Table 1, the median (IQR) age of respondents was 30 (24–41), and 49% of the respondents were female; these did not differ by re-settlement location. The majority of both in camp (70%) and out of camp (73%) respondents were married at the time of the survey. In camp and out of camp respondents had similar rates of illiteracy (46 and 44% respectively), education levels, and main occupations prior to the IS attack (Table 1). Employment differed between the two groups (*p* = 0.002) after the IS attack with respondents living inside camps having higher rates of unemployment (29% versus 20%) and lower rates of being self-employed (14% versus 16%). Income also differed between the two groups (*p* = 0.02), with respondents living in camps more likely to have incomes under 300,000 IQD per month (65% versus 55%).

**Table 1:** Characteristics of 1256 Yazidi internally displaced persons included in this study

		All ***n*** = 1256	In Camp ***n*** = 794 (63%)	Out of Camp ***n*** = 462 (37%)	***p***-value
**Age, years**		30 (24–41)	30 (24–40)	31 (24–42)	0.26
**Female**		49%	49%	48%	0.80
**Marital Status**	**Married**	71%	70%	73%	0.46
	**Widowed**	2%	3%	2%	
	**Divorced**	0%	1%	0%	
	**Single or never married**	26%	27%	25%	
**Illiterate**		45%	46%	44%	0.35
**Education level**	**None**	42%	42%	41%	0.17
	**Some primary**	15%	16%	14%	
	**Finished primary**	11%	10%	12%	
	**Intermediate**	19%	16%	16%	
	**High school or above**	13%	11%	17%	
	**Other**	0%	0%	0%	
**Main occupation before ISIS attack**	**Farming/Livestock**	13%	14%	12%	0.65
	**Government employee**	4%	3%	4%	
	**Self-employed**	19%	20%	18%	
	**Police/army**	4%	5%	4%	
	**Student**	22%	22%	22%	
	**Housewife**	30%	29%	31%	
	**Unemployed**	5%	5%	5%	
	**Other**	3%	2%	4%	
**Employment after ISIS attack**	**Farming/Livestock**	2%	2%	3%	0.002
	**Government employee**	4%	4%	4%	
	**Self-employed**	16%	14%	16%	
	**Police/army**	3%	3%	3%	
	**Student**	7%	7%	7%	
	**Housewife**	38%	38%	38%	
	**Unemployed**	26%	29%	20%	
	**Other**	4%	3%	7%	
**Current income**	**< 300,000 IQD per month**	61%	65%	55%	0.02
	**Between 300,000–600,000 IQD per month**	20%	18%	25%	
	**> 600,000 IQD per month**	8%	7%	9%	
	**No response/Other**	11%	11%	11%	

*Median (IQR)

### Exposure to violence

Complete data on exposure to 14 trauma events were assessed among respondents (Table 2).

**Table 2:** Comparison of exposure to violence for IDPs living in camp and out of camp

		All ***N*** = 1256	In Camp ***n*** = 794 (63%)	Out of Camp ***n*** = 462 (37%)	***p***-value
**Witness killing or murder**	**Exposed**	23%	27%	18%	< 0.001
	**No response**	0%	0%	0%	
**Witness beating or torture to head or body**	**Exposed**	15%	12%	17%	0.017
	**No response**	0%	0%	0%	
**Witnessed rape/sexual abuse**	**Exposed**	3%	3%	3%	0.64
	**No response**	0.1%	0.1%	0%	
**Were threatened with death**	**Exposed**	30%	31%	27%	0.21
	**No response**	0%	0%	0%	
**Were starved/lack of nutrition**	**Exposed**	69%	70%	68%	0.33
	**No response**	0%	0%	0%	
**Combat Situation**	**Exposed**	78%	81%	73%	0.002
	**No response**	0%	0%	0%	
**Forced Evacuation**	**Exposed**	56%	57%	54%	0.40
	**No response**	0%	0%	0%	
**Beating and/or mutilation to the body**	**Exposed**	5%	5%	4%	0.23
	**No response**	0%	0%	0%	
**Rape or other types of sexual abuse or humiliation**	**Exposed**	2%	3%	2%	0.52
	**No response**	0%	0%	0%	
**Disability as result of combat situation or landmine**	**Exposed**	4%	4%	3%	0.22
	**No response**	0%	0%	0%	
**Torture**	**Exposed**	16%	18%	12%	0.004
	**No response**	0%	0%	0%	
**Imprisonment**	**Exposed**	8%	8%	6%	0.16
	**No response**	0%	0%	0%	
**Being kidnapped/abducted for over a week**	**Exposed**	3%	3%	3%	0.69
	**No response**	0%	0%	0%	
**Other forced separation from family members**	**Exposed**	6%	6%	5%	0.52
	**No response**	0%	0%	0%	

Exposure to violence was highly prevalent among the respondents during ISIS control of their governorate. Seventy-eight percent of respondents had been exposed to a combat situation, and 69% had experienced starvation/lack of nutrition. Fifty-six percent of respondents had experienced forced evacuation, 30% had been threatened with death, 23% had witnessed a killing or murder, and 16% had been tortured, and 15% had witnessed torture. Smaller percentages of respondents had been imprisoned (8%), had forced separation from family members (6%), experienced beating and/or mutilation to body (5%), developed disability as result of combat situation or landmine (4%), witnessed rape/sexual abuse (3%), been kidnapped/abducted for over a week (3%), or experienced rape or other types of sexual abuse/humiliation (2%).

Reported exposure to violence differed between Yazidi IDPs in camps and those out of camps. When looking at total past exposure, IDPs living in camps were more likely to have witnessed killing or a murder (OR 1.69, 95% CI 1.27–2.25 *p* < 0.001), to have witnessed beating or torture to head or body (OR 1.50, 95% CI 1.07–2.09 p = 0.02), to have experienced a combat situation (OR 1.52, 95% CI 1.16–2.00 p = 0.002), and to have been victims of torture (OR 1.61, 95% CI 1.16–2.26 *p* = 0.004). Rates of other exposures to violence were similar between the two groups.

### Sense of community and trust

Perceptions about trust and community are shown as percentages in Table 3.

**Table 3:** Comparison of social cohesion measures between IDPs in camps versus out of camps

		All ***n*** = 1256	In Camp ***n*** = 794 (63%)	Out of Camp ***n*** = 462 (37%)	***p***-value
**Relationship with family**	Very Bad	< 1%	< 1%	< 1%	0.50
	Bad	1%	1%	1%	
	Average	3%	3%	3%	
	Good	39%	37%	42%	
	Very Good	56%	58%	53%	
	No Response	0%	0%	0%	
**Relationship with friends and neighbors**	Very Bad	< 1%	< 1%	0%	0.08
	Bad	< 1%	< 1%	< 1%	
	Average	2%	2%	2%	
	Good	47%	45%	50%	
	Very Good	50%	52%	46%	
	No Response	< 1%	0%	< 1%	
**Relationship with community**	Very Bad	< 1%	< 1%	< 1%	0.77
	Bad	1%	1%	2%	
	Average	9%	9%	7%	
	Good	54%	53%	54%	
	Very Good	36%	36%	37%	
	No Response	0%	0%	0%	
**Relationship with people from other ethnic groups**	Very Bad	1%	1%	2%	0.03
	Bad	5%	5%	6%	
	Average	12%	14%	8%	
	Good	53%	54%	53%	
	Very Good	28%	26%	31%	
	No Response	< 1%	< 1%	< 1%	
**Relationship with people from other religious groups**	Very Bad	< 1%	< 1%	< 1%	0.02
	Bad	5%	5%	5%	
	Average	10%	12%	6%	
	Good	55%	55%	56%	
	Very Good	29%	27%	32%	
	No Response	< 1%	< 1%	< 1%	
**Your relationship with people from your ethnic group**	Very Bad	0%	0%	0%	0.65
	Bad	< 1%	< 1%	< 1%	
	Average	4%	5%	4%	
	Good	56%	55%	58%	
	Very Good	39%	40%	38%	
	No Response	0%	0%	0%	
**Your relationship with people from your religious group**	Very Bad	< 1%	< 1%	0%	0.30
	Bad	< 1%	< 1%	< 1%	
	Average	4%	4%	3%	
	Good	53%	51%	57%	
	Very Good	42%	44%	39%	
	No Response	< 1%	< 1%	0%	
**How much do you trust the people in your current location**	Not at all	3%	3%	2%	0.01
	A little bit	6%	6%	7%	
	Moderately	19%	21%	16%	
	Quite a bit	42%	38%	47%	
	Extremely	30%	32%	27%	
	Don’t Know	< 1%	< 1%	0%	
**Feel safe where you are staying now**	Yes	94%	93%	97%	0.01
	Missing	1%	2%	< 1%	
**In place you consider home**	Yes	69%	68%	72%	0.10
	Missing	1%	2%	< 1%	

When asked about sense of safety in their current location, 94% of respondents reported that they felt safe in the location that they were residing, and 69% of them considered their current residence their home. Seventy-two percent of respondents responded that they trust the people in their current location “Quite a bit” or “Extremely.” Overall, there were high rates of positive relationships with family (95% “Good” or “Very Good”), with friends and neighbors (97% “Good” or “Very Good”), with the community in general (90% “Good” or “Very Good”), with people from other ethnic groups (81% “Good” or “Very Good”) and people from other religious groups (84% “Good” or “Very Good”). In an aggregated score looking at perception of relationships, ranging from 1(lowest) to 29 (highest), the median score was 23 (IQR 22–27).

Perceptions of community and relationships differed between the two groups. Respondents residing out of camps were more likely to trust people in their current location “Quite a Bit” (47% versus 38%) but less likely to trust people “Extremely” (27% versus 32%, *p* = 0.01). Respondents residing out of camps were nearly 2 times more likely to feel safe in the location of their residence (OR 1.99, 95% CI 1.08–3.67, *p* = 0.03). Out of camp respondents were more likely to report “Very Good” relationships with people from any other ethnic group (31% versus 26%, *p* = 0.049) or “Very Good” relationship with people from any other religious group (31% versus 27%, *p* = 0.04). In an aggregated score of social relationships, there was no difference between those residing in camps versus out of camps (*p* = 0.79).

### Quality of life and access to services

Perceptions regarding access to basic services varied widely depending on the service or commodity. Fifty-one percent of respondents reported “Good” or “Very good” access to water, 65% reported “Average” or above access to food, 57% reported “Average” or above access to education if needed, and 57% reported “Average” or above housing quality. However, respondents were less satisfied regarding access to healthcare, ability to find employment, and access to administrative services, with 50, 88, and 68% respectively of respondents reporting “Bad” or “Very bad” access. The median number of medium assets was 3 (out of 3), and the median number of extended assets was 4 (out of 8).

The distribution of responses also differed significantly by group, with more people living out of camps describing their housing as “Very bad” compared to those in camps (18% versus 12%, *p* = 0.02). More respondents living out of camps also reported having “Very bad” access to food (14% versus 5%, *p* < 0.001), access to healthcare (19% versus 11%, *p* = 0.001), and access to education (16% versus 10%, *p* = 0.02). Both groups reported high rates of access to water (*p* = 0.61), and poor ability to find work/employment (*p* = 0.17). There was no difference in number of medium assets (*p* = 0.07) or extended assets (*p* = .26) between the groups.

### Associations between accommodation and symptoms of distress

Respondents overall reported high levels of distress symptoms. The median (IQR) distress score, on a scale of 0–88, was 45 (28–57) among all respondents. Sixty-nine percent of respondents had a score greater than or equal to 33. Among IDPs living in camps, their median (IQR) distress score was 48 (31–60), and among those living out of camps median (IQR) distress score was 40 (24–53). Seventy-three percent of IDPs in camps and 64% of those out of camps had scores greater than or equal to 33 (OR 1.50, 95% CI 1.17–1.91 *p* = 0.001).

In univariable linear regression, residing in a camp was associated with an average of a 7.7 point (19%) increase in distress score from a predicted mean distress score of 38.75 (95% CI 5.49–9.91 *p* < 0.001); a complete list of results from univariable regression can be found in Table 4.

**Table 4:** Logistic regression

	Univariable Coefficient ***P***-value	Multivariable Coefficient
**Staying in camps**	−7.70 < 0.001	−7.15 < 0.001
**Exposure to Violence**		
***Witness killing or murder***	4.77	
	< 0.001	
***Witness beating or torture to head or body***	6.90	3.7 0.01
	< 0.001	
***Witnessed rape/sexual abuse***	13.97	
	< 0.001	
***Were threatened with death***	4.15	
	0.001	
***Were starved/lack of nutrition***	8.76	7.73 < 0.001
	< 0.001	
***Combat Situation***	2.57	
	0.05	
***Forced Evacuation***	4.60	−2.37
	< 0.001	0.047
***Beating and/or mutilation to the body***	8.70	
	0.001	
***Rape or other types of sexual abuse or humiliation***	10.38	
	0.005	
***Disability as result of combat situation or landmine***	11.12	7.25
	< 0.001	0.008
***Torture***	8.05	3.94
	< 0.001	0.009
***Imprisonment***	4.97	
	0.01	
***Being kidnapped/abducted for over a week***	16.38	
	< 0.001	
***Other forced separation from family members***	14.17	7.75
	< 0.001	0.001
**Social Cohesion**		
***Relationship with people from any other ethnic group***	1.74	
	0.008	
***Relationship with people from any other religious group***	2.74	3.40
	< 0.001	< 0.001
***Amount of trust in people in current living situation***	1.33	1.35
	0.017	0.017
***Feeling of safety in current living situation***	−7.89	−6.44
	0.002	0.009
**Housing**	−1.23	
	0.11	
**Access to food**	1.76	2.80
	0.001	< 0.001
**Access to healthcare**	−1.65	−1.82
	0.001	0.001
**Ability to find work/employment**	−1.46	
	0.04	
**Access to education if needed**	−1.06	−1.48
	0.04	0.002
**Access to administrative services**	0.71	
	0.17	
**Current Unemployment**	2.26	3.82
	0.07	0.002
**Current Income**	0.01	
	0.77	

After conducting backwards stepwise regression with all variables that were associated with distress score with a *p* < 0.2, staying in a camp remained significantly associated with an increased in distress score on average of 7.2 points (*p* < 0.001), after adjusting for witnessing beating or torture, experiencing starvation/lack of nutrition, forced evacuation, disability as result of combat situation or landmine, torture, other forced separation from family members, quality of relationship with people from any other religious group, amount of trust in people in current living situation, feeling of safety in current living situation, access to food, access to healthcare, access to education, and current unemployment.

## Discussion

Our study found a high prevalence of distress symptoms among Yazidi IDPs living in northern Iraq, with 69% of respondents meeting a distress symptom threshold that is highly predictive of PTSD. We also found that post-displacement settlement setting (in camp compared to out of camp) was significantly associated with higher distress symptom scores, with IDPs in camps predicted to have a 19% higher mean score compared to IDPs out of camps. Even though IDPs in camps had higher rates of exposure to violence, this difference in mean distress symptom scores remained significant even after controlling for exposure to violence, social cohesion, unemployment, and access to services.

Moreover, despite IDPs out of camps having a lower prevalence of distress symptoms, they reported worse access to many important services, including access to food, healthcare, and education. Our findings are consistent with a survey conducted in 2017 as part of the Mixed Migration Platform, which found that IDPs living in private accommodations had a more negative perception of access to services compared to their counterparts living in camps [29]. Difficulties in accessing basic services and a higher rate of respondents reporting being very dissatisfied with their housing reflect the challenges of the decentralized nature of safety net services for displaced persons in out of camp settings.

These findings raise several important questions. First, the association between lower distress scores and out of camp accommodation, even after controlling for exposure to violence, may suggest that post-displacement accommodation could be viewed as an intervention in itself to reduce the severity of post-trauma sequelae. While there is a growing body of research demonstrating the importance of the built environment and physical space in impacting well-being, this is still poorly studied among displaced populations [33, 34]. Our research lays the necessary foundation for investigating this question further. Second, we observed this association despite IDPs reporting poorer access to basic services and greater dissatisfaction with their housing in out of camp settings. This highlights the ongoing conflict between prioritizing access to basic services in a centralized setting such as a camp, while upholding the dignity, autonomy, resiliency, and privacy of displaced persons. It also may suggest the relative importance of factors related to self-efficacy, such as independence and dignity, on mental health and alleviating the sequelae of trauma. Finally, our findings highlight the challenge of reduced visibility for IDPs residing in non-camp settings and how it can impede their ability to receive assistance and services. In fact, the Internal Displacement Monitoring Centre (IDMC) reported that IDPs in gathered settings, such as camps, were twice as likely to receive assistance and protection from national authorities and humanitarian actors compared to those in dispersed settings [28, 35]. Exploring creative development solutions where support can be provided to IDPs who are not in formal camp settings without overburdening host communities will be a challenge, but could be effective in addressing any pre-existing structural issues faced by host communities. Such solutions could consequently facilitate and strengthen a greater link between humanitarian actors and vulnerable populations while also promoting development for the host communities.

Our findings also highlight an important issue within an ongoing discussion around collective trauma. Many have argued that traditional Western psychological models are inadequate in the international context, and instead argue for more trans-cultural and post-modern approaches to psychological care [36]. Western models have also been critiqued for their internalized problem discourse, putting the burden of trauma on the individual and absolving society from its collective responsibility. Such an internalized problem discourse fails to acknowledge the ways in which interventions aimed at improving the socioeconomic and living conditions may be a strategy for improving mental health. Our research provides an important groundwork for thinking about such strategies.

Our study has some limitations. First, while we did not find any demographic differences among Yazidi IDPs resettled in camps versus out of camps, we do not know whether the location of post-displacement resettlement is impacted by where they were resided within Nineveh governorate pre-displacement as we did not ascertain this information. In addition, we lacked information regarding the quality of housing, such as number of individuals sharing a unit and size of unit. Furthermore, though we had a holistic survey looking at exposure to violence, it is possible that we missed exposures or did not adequately assess severity of exposure, which could have differed by post-displacement accommodation. Furthermore, we did not have information regarding time from exposure to violence, which could have impacted distress scores and potentially differed between IDPs settled in camp versus out of camp.

Despite these limitations, our study lays important groundwork to begin investigating alternatives to camp-like settlements for displaced populations to improve their well-being and positive long-term outcomes. As the humanitarian community begins to shift towards new models of emergency and long-term housing, it will also be important to understand the potential and limitations of more decentralized models, and identify effective methods to maintain access to basic services while improving conditions for both displaced populations and host communities.

## Data Availability

The data that support the findings of this study are available from Phuong Pham but restrictions apply to the availability of these data, which were used under license for the current study, and so are not publicly available. Data are however available from the authors upon reasonable request and with permission of Phuong Pham, ppham@hsph.harvard.edu.

## Abbreviations

IDPs: Internally displaced persons
IES-R: Impact of Event Scale-Revised
IOM: International Organization for Migration
IQR: Interquartile ranges
IS: Islamic State
KRI: Kurdistan Region of Iraq
PTSD: Post-traumatic stress disorder
UNHCR: United Nations High Commissioner for Refugees
